# Quantitation of Enterovirus A71 Empty and Full Particles by Sedimentation Velocity Analytical Ultracentrifugation

**DOI:** 10.3390/v16040573

**Published:** 2024-04-08

**Authors:** Anna Yang, Yun Luo, Jie Yang, Tingbo Xie, Wenhui Wang, Xin Wan, Kaiwen Wang, Deqin Pang, Dongsheng Yang, Hanyu Dai, Jie Wu, Shengli Meng, Jing Guo, Zejun Wang, Shuo Shen

**Affiliations:** 1 Wuhan Institute of Biological Products Co., Ltd., No. 1 Huangjin Industrial Park Road, Wuhan 430207, China13163255896@163.com (J.Y.); 18995586191@163.com (S.M.);; 2 National Engineering Technology Research Center of Combined Vaccines, No. 1 Huangjin Industrial Park Road, Wuhan 430207, China; 3 National Key Laboratory for Novel Vaccines Research and Development of Emerging Infectious Diseases, No. 1 Huangjin Industrial Park Road, Wuhan 430207, China; 4 Hubei Provincial Vaccines Technology Innozation Center, No. 1 Huangjin Industrial Park Road, Wuhan 430207, China; 5 The Research Core Facilities for Life Science (HUST), College of Life Science and Technology, Huazhong University of Science and Technology, Luoyu Road, Wuhan 430074, China

**Keywords:** SV-AUC, EV71 inactivated vaccine, quality control, empty particles, full particles

## Abstract

The enterovirus A71 (EV71) inactivated vaccine is an effective intervention to control the spread of the virus and prevent EV71-associated hand, foot, and mouth disease (HFMD). It is widely administered to infants and children in China. The empty particles (EPs) and full particles (FPs) generated during production have different antigenic and immunogenic properties. However, the antigen detection methods currently used were established without considering the differences in antigenicity between EPs and FPs. There is also a lack of other effective analytical methods for detecting the different particle forms, which hinders the consistency between batches of products. In this study, we analyzed the application of sedimentation velocity analytical ultracentrifugation (SV-AUC) in characterizing the EPs and FPs of EV71. Our results showed that the proportions of the two forms could be quantified simultaneously by SV-AUC. We also determined the repeatability and accuracy of this method and found that both parameters were satisfactory. We assessed SV-AUC for bulk vaccine quality control, and our findings indicated that SV-AUC can be used effectively to analyze the percentage of EPs and FPs and monitor the consistency of the process to ensure the quality of the vaccine.

## 1. Introduction

Hand, foot, and mouth disease (HFMD) is caused by enteroviruses, mainly in infants and children below five years old [1,2,3]. It caused epidemics worldwide, particularly in Asian and Pacific regions, after its discovery in California in 1969 [4,5,6,7,8]. Enterovirus 71 (EV71) is one of the main causative agents of HFMD among various enteroviruses. It can lead to severe complications, including neurological disorders and even death [9]. Three EV71 inactivated vaccines are commercially available in China [10,11,12,13,14,15]. EV71 is an icosahedral, non-enveloped virus with a single-stranded, positive-sense RNA genome. It is classified within the *Enterovirus* genus of the *Picornaviridae* family and may cause the infection of the central nervous system (CNS) in children, leading to meningitis, encephalitis, brain imaging abnormalities, and long-term neurodevelopmental sequelae [16,17,18]. When cultured in mammalian cells, enteroviruses can simultaneously generate two major types of stable particles: full particles (FPs), which contain the RNA genome, and immature empty particles (EPs), which lack the RNA genome. The FP contains VP1, VP2, VP3, VP4, and viral RNA, while the EP contains VP0, VP1, and VP3 [19,20,21]. VP0 is an uncleaved precursor of VP4 and VP2. The final step in the maturation of most picornaviruses is the cleavage of VP0 into VP2 and VP4. However, the mechanism underlying the cleavage of VP0 remains poorly understood [22]. Thus, the mechanism of the formation of EPs and FPs is not clear.

The EPs and FPs of EV71 have different antigenic and immunogenic properties and may affect the quality control and process control of vaccine production. Regulatory agencies recommend evaluating or testing the ratio between the two particle types [23,24]. Therefore, a sensitive and accurate method needs to be developed to quantify the proportions of EPs and FPs. Poliovirus also exists as two particle forms, which are commonly differentiated using the D and C antigen-specific antibody enzyme-linked immunosorbent assay (ELISA) [25,26]. The D antigen stimulates a stronger neutralizing antibody response than the C antigen. The ELISA is highly sensitive and specific; however, screening suitable antibodies for the specific quantitative detection of EP and FP antigens is time- and labor-consuming. Several other methods have been evaluated for their abilities to assess the capsid content in Adeno-associated viruses (AAVs). These methods include ultraviolet (UV) absorbance [27], ion-exchange chromatography [28], sedimentation velocity analytical ultracentrifugation (SV-AUC) [29], charge detection mass spectrometry (CDMS) [30], cryo-electron microscopy (cryo-EM) [31], size exclusion chromatography coupled with multiangle light scattering (SEC-MALS) [32], and analytical band centrifugation (ABC) [33]. These methods have their own pros and cons. UV absorbance, ion-exchange chromatography, and SEC-MALS have high throughput with easy accessibility. CDMS has low material requirements and can obtain more information on the mass and charge of the capsid. Cryo-EM can provide the capsid shape and size through direct visualization. ABC does not require additional reagents. Although these methods are still used, all of them have certain limitations, such as low throughput, the indirect measurement of the capsid content, potential interference from impurities, etc. [34]. Among these methods, SV-AUC is the gold standard for characterizing the full particle content of AAVs [29,34,35].

The SV-AUC method can differentiate EPs and FPs based on their buoyant densities. The data are obtained by directly modeling the sedimentation boundary profiles generated from Rayleigh interference optics (IF) or UV detection systems, using the SEDFIT software (https://sedfitsedphat.github.io/) [36]. During detection, samples remain in a solution state, avoiding interactions with other substances; sample detection does not require additional labeling reagents or antibodies. Particle separation can be monitored in real-time during centrifugation [36,37,38].

In this study, we investigated whether SV-AUC can be used to analyze the relative content of EPs and FPs in EV71. The results showed that the method had satisfactory accuracy and precision and could withstand different detection temperatures. The results obtained by the ultraviolet detector and the IF system were very similar. To assess whether the method was suitable for routine inspection in quality control, we also evaluated the stability of the production process. Overall, our findings showed that SV-AUC is an effective technique for evaluating and controlling the heterogeneity of virus particles in inactivated vaccines.

## 2. Materials and Methods

### 2.1. EV71 EP and FP Production and Purification

African green monkey kidney (Vero) (WIBP cell bank) cells were cultured in complete Dulbecco’s modified Eagle medium (DMEM, Thermo Fisher, Waltham, MA, USA) with 10% (*v*/*v*) newborn calf serum (NCS). EV71 strain AHFY087VP5 (genotype C4) was inoculated in Vero cells in a 10-layer cell factory at a multiplicity of infection (MOI) of 0.01 CCID50/mL per cell in DMEM in the absence of NCS and then cultured in a 5% CO_2_–air incubator at 35 °C for 3 days before harvest. The cell factory was freeze-thawed three times. The cell debris were removed by centrifugation at 4000× *g* in a Beckman JA10 rotor (Beckman Coulter, Indianapolis, IN, USA) for 30 min at 4 °C. The supernatant was concentrated to one-tenth the harvest volume by using a 100 kDa tangential flow filter capsule (Sartorius, Göttingen, Germany). The concentrate was centrifuged at 4 °C through a sucrose cushion (30% (wt/vol)) in a Beckman SW28 rotor (Beckman Coulter, Indianapolis, IN, USA) at 103,745× *g* for 3 h. The pellets were resuspended in phosphate-buffered saline (PBS), pH 7.2, at 4 °C overnight. Then, EPs and FPs were isolated and purified via cesium chloride (CsCl) density gradient (1.30 g/mL) centrifugation. The centrifugation was conducted at 48,000 rpm at 4 °C for 24 h in the Beckman Optima TM L-90K Ultracentrifuge (Beckman Coulter, Indianapolis, IN, USA) equipped with a 90Ti rotor. Then, the buffer was replaced into PBS, pH 7.2, using an Amicon Ultra-15 centrifuge tube (Ultracel-10 kDa) (Merck KGaA, Darmstadt, Germany), and the centrifugation conditions are consistent with those used for concentration.

### 2.2. Purification of EV71 Vaccine Bulks

EV71 vaccine bulks were manufactured by the Wuhan Institute of Biological Products Co., Ltd. The process of preparing the vaccine bulks included the following steps: the culturing of Vero cells and virus propagation, the harvesting of cells, the removal of cell debris, microfiltration, ultrafiltration, gel filtration chromatography, ion-exchange chromatography, and formaldehyde inactivation. (The details for the purification of vaccine bulks are confidential information of the Wuhan Institute of Biological Products Co., Ltd., so they cannot be shared.)

### 2.3. Characterization of EV71 EPs and FPs

The purified EV71 particles were analyzed via SDS-PAGE. The virus proteins were separated on a 4–20% gradient polyacrylamide gel (Genscript, SurePAGE™, Nanjing, China) at 80 mA for an hour and a half. Carbon-coated copper grids (200 mesh) were covered on the surface of 20 μL EV71 particles for 10 min, stained with 20 μL of 1% phosphotungstic acid, pH 7.0, for 10 min, and the excess droplet was removed and dried. The morphological characteristics of the EV71 particles were examined by a transmission electron microscope (TEM) (Thermo Fisher, Hillsboro, OR, USA).

### 2.4. Sample Preparation and SV-AUC Data Acquisition

The sample’s absorbance signal was determined by Nanodrop (Thermo Fisher, Waltham, MA, USA). The sample’s concentration was adjusted to a certain concentration (an OD of between 0.6 and 1.0) further concentrated by using an Amicon Ultra-15 centrifuge tube (Ultracel-10 kDa) (Merck KGaA, Darmstadt, Germany) or direct dilution with PBS, pH 7.2. The samples were assessed using a Beckman Coulter ProteomeLab XL-I instrument (Beckman Coulter, Indianapolis, IN, USA) equipped with a four-hole An-60Ti rotor at 15,000 rpm at 20 °C or 4 °C. Approximately 400 µL of samples and blank buffer was loaded into the sample and reference sectors, respectively. The absorbance data were collected for scanning across a radius range of 5.9 cm to 7.15 cm. The absorbance (230, 260, and 280 nm) and IF were used to simultaneously record the radial concentration.

### 2.5. SV-AUC Data Analysis

The SV-AUC data were analyzed using the SEDFIT software to show the sedimentation coefficient distribution [36,39,40]. The C(S) was calculated by choosing “Continuous Sedimentation Coefficient Distribution C(S)”. The C(S) parameters were as follows: resolution = 100S, confidence level = 0.95, s min = 40, s max = 250, partial spec. volume = 0.73, buffer density = 1.00, and buffer viscosity = 0.01002. The model fitted the data to the Lamm equation, and the resulting size distribution was a sedimentation coefficient distribution, where the area under each peak was proportional to the concentration in fringes or the optical density (OD). The results of the AUC analysis were plotted as the normalized differential C(S) and sedimentation coefficient. The FP percentage was calculated using the equation

(1) FP% = c(150S)/[c(80S) + c(150S)],

where c(80S) and c(150S) represent the c-value (the area under each peak) for EPs and FPs, respectively.

### 2.6. Statistical Analysis

The linear regression correlation and Pearson’s correlation coefficient were determined using GraphPad Prism 8.0 (GraphPad; San Diego, CA, USA). All differences were considered to be statistically significant at *p* < 0.05.

## 3. Results

### 3.1. Preparation and Characterization of EV71 EPs and FPs 

**Figure 1:**
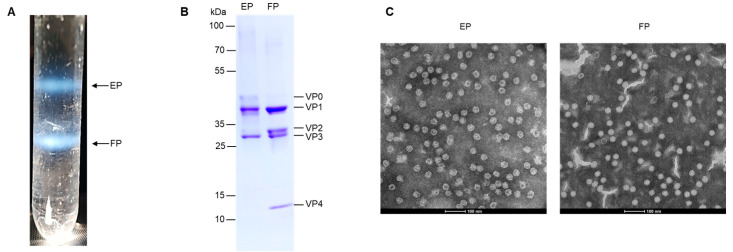
The purification and characterization of EV71 EPs and FPs. (**A**) The positions of EPs and FPs are indicated in the CsCl gradient (equilibrium at a starting density of 1.30 g/mL). (**B**) The purified EPs and FPs were analyzed by SDS-PAGE. VP0, VP1, VP2, VP3, and VP4 are labeled on the right, and the molecular weight markers in kDa are indicated on the left. (**C**) The electron microscope’s examination of inactivated EPs and FPs; scale bar: 100 nm.

The EV71 EPs and FPs were isolated and purified via CsCl density gradient centrifugation. After centrifugation, the virus particles were separated into two bands (Figure 1A). The composition of the structural proteins of the EPs and FPs was analyzed by SDS-PAGE (Figure 1B). The EP consisted of VP0, VP1, and VP3 and the FP consisted of VP1, VP2, VP3, and VP4. The identity of EPs and FPs was confirmed by TEM examination (Figure 1C). The EP and FP were about 30 nm in diameter. EPs showed a void due to the absence of nucleic acid and the increased permeability, which allowed Cs ions to enter the particles. The FP showed a native conformation and was more compact than the EP.

### 3.2. SV-AUC for EV71 EPs and FPs

**Figure 2:**
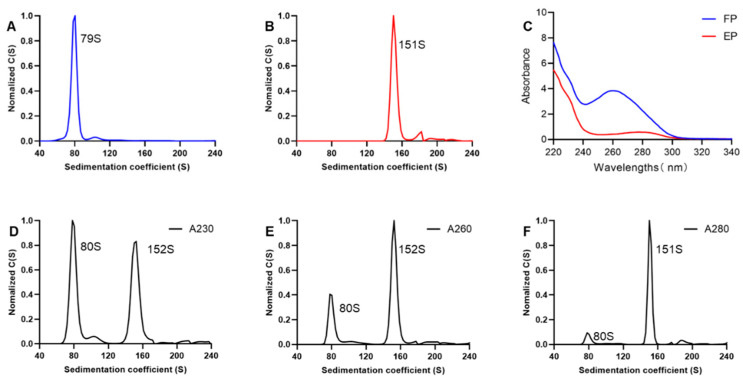
SV-AUC for EV71 EPs and FPs. (**A**,**B**) The sedimentation coefficient distribution plot of EV71 EPs and FPs. (**C**) UV absorption spectra of EPs and FPs. (**D**–**F**) The distribution maps of settlement coefficients were obtained by analyzing at detection wavelengths of 230 nm, 260 nm, and 280 nm, respectively. The x-axis represents the sedimentation coefficient of the sample component, and the y-axis represents the sample concentration distribution function with the sedimentation coefficient used as a variable. The particle settlement coefficients corresponding to the peaks are marked next to the peaks.

The boundary sedimentation velocity of EPs and FPs was assessed by SV-AUC. The detection wavelength of the UV detector was set at 280 nm. SV-AUC is based on fluid dynamics theory. Under high-speed rotation, solute molecules in the sample move toward the bottom of the cell. The UV absorption signals of the entire sample cell at different positions and times are monitored by the detector. Using the SEDFIT software, the results of the AUC analysis were plotted as normalized differential coefficient distribution values C(S) versus the sedimentation coefficient (Figure 2A,B). The sedimentation coefficients of FPs and EPs were 151S and 79S, respectively. The sedimentation coefficients of EPs were consistent with EV71-VLP, as reported in another study [41]. Since the FP contains nucleic acids, its sedimentation coefficient is significantly greater than that of the EP. Therefore, the two particle types can be distinguished based on their different sedimentation coefficients.

When an ultraviolet detector is used for detection, the c-value is proportional to the sample absorbance. Particles with the same protein concentration may exhibit different absorbance values at the same wavelength due to variations in their absorption spectra. Therefore, the detection wavelength needs to be optimized when an ultraviolet detector is used for data analysis. Purified EPs and FPs were diluted to equivalent protein concentrations, and their ultraviolet absorption spectra were characterized using a spectrophotometer. The maximum absorption for the EP, which was mostly composed of viral structural proteins, occurred at 280 nm, consistent with proteins. In contrast, the maximum absorption for FPs was 260 nm, which indicated that the viral genomic nucleic acids present in FPs strongly affected their absorption profiles (Figure 2C). Based on the findings of Khasa et al., 230 nm was used as the detection wavelength, as it could eliminate this discrepancy [33]. Therefore, after mixing equal quantities of purified EPs and FPs, the blended samples were evaluated at three different detection wavelengths (230, 260, and 280 nm), and the fractional compositions of EPs and FPs were compared at the three wavelengths for the same sample (Figure 2D–F). Two particle forms were differentiated based on their sedimentation coefficients, with EPs at 80S and FPs at 151S. The c-value calculated using the SEDFIT software indicated the peak area, which was proportional to the protein concentration. The percentage of FPs at 260 nm and 280 nm substantially exceeded theoretical values, while the percentage of FPs at 230 nm aligned with the anticipated ratios (Table S1). Therefore, 230 nm was selected as the optimal detection wavelength for subsequent analyses.

### 3.3. Comparison of Interference and Ultraviolet Detection

**Figure 3:**
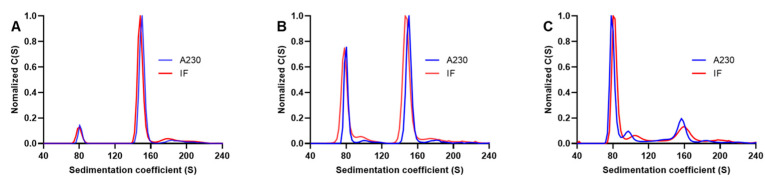
A comparison of interference and ultraviolet detection. (**A**–**C**) The sedimentation distribution plots were obtained by UV absorbance at 230 nm and IF with different proportions of EP and FP. (**A**–**C**) included samples with low, medium, and high proportions of EP, respectively. The results obtained by the ultraviolet (at 230 nm) and IF detection methods are shown in the same graph for comparison.

Besides the ultraviolet detector, AUC can also be equipped with a Rayleigh interference optics system. Therefore, the differences in detection between the two systems were also investigated. Three mixtures with different ratios of purified EPs and FPs were prepared. The detection was simultaneously performed using ultraviolet absorbance (230 nm) and IF. The results showed that the normalized peak graphs and sedimentation coefficient values were similar in the two detection methods (Figure 3A–C), and the relative percentage composition of the particles was close to 1 (Tables S2 and S3). The IF system detected the sample concentration based on the differences in the refractive indices between the reference and sample solutions. The unique nucleic acids present in FP had a negligible effect on the concentration measurements by interference. Therefore, the consistent results from the two detection methods indicated that using 230 nm could significantly reduce the effect of the nucleic acids in FPs on the measurements. Compared to the ultraviolet detector, the interference system yielded considerably lower c-values for samples at the same concentration with 3–4-fold differences. This discrepancy suggested that the interference detector had lower sensitivity than the ultraviolet detector. Thus, higher sample concentrations were needed for interference detection. Interference detection is non-selective, with a lower tolerance for mismatch between sample and reference buffer compositions, which makes the results more susceptible to reference buffer effects. It also demands higher sample purity [34]. However, biological samples after purification usually have relatively low protein concentrations and less than 100% purity. Therefore, we selected 230 nm as the detection wavelength for subsequent experiments.

### 3.4. Performance of SV-AUC for Quantifying EV71 EPs and FPs

**Figure 4:**
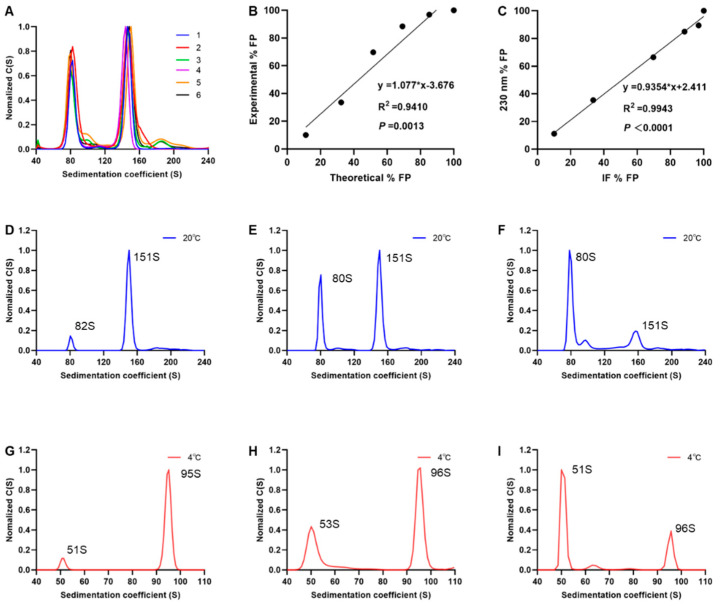
Performance of SV-AUC for quantifying EV71 EPs and FPs. (**A**) Precision verification results of SV-AUC. Sedimentation distribution plots of 6 times were superimposed. Accuracy verification results of SV-AUC. (**B**) Linear regression analysis of detected and theoretical FP proportions. (**C**) Linear regression analysis of A230 and IF detection results. (**D**–**I**) Comparison of different detection temperatures. Sedimentation distribution plots obtained by A230 of samples with different mixed proportions of EPs and FPs. From left to right are samples with low, medium, and high proportions of EPs, respectively.

To evaluate whether the SV-AUC technique showed repeatability, the EV71 vaccine bulk was characterized using SV-AUC. For each assay, three AUC cells were sequentially loaded. Two discrete experiments were conducted at different time points, and six datasets were generated (Figure 4A). The mean of the six assay results was evaluated, and the fractional composition showed 42.50% of EPs and 57.50% of FPs. The coefficients of variation (CVs) were 6.11% and 4.52% for EPs and FPs, respectively (Table S4). The sedimentation coefficient CV was uniformly below 2.50%, indicating that the method was stable.

To evaluate the accuracy of SV-AUC for characterizing the proportions of EV71 EPs and FPs, six samples with different ratios of purified EPs and FPs were prepared and analyzed by SV-AUC at 230 nm. The results of the regression analysis showed a highly linear relationship between the detected and theoretical FP proportions (R^2^ = 0.941). The slope of the regression equation was 1.077 (Figure 4B; close to 1), which indicated a strong correlation between the detected and theoretical values. The particle proportions of the mixed samples were also simultaneously assessed by IF. The linear regression analysis of the detection results showed a correlation coefficient of 0.9943 between the two methods (Figure 4C; *p* < 0.0001). The differences between the two methods ranged from −1.08% to 7.47% (Table S5), indicating high consistency between the ultraviolet (230 nm) and IF detection methods. 

Temperature changes can affect the rate and time of the sedimentation of the particles and the percentage of EPs and FPs. Therefore, the differences in the detection of SV-AUC at two different temperatures were also investigated. Three samples were prepared using different ratios of EPs and FPs and analyzed using SV-AUC at 4 °C and 20 °C. The results showed that the sedimentation coefficients of EPs and FPs measured at 4 °C were 52S and 96S, respectively, while the coefficients at 20 °C were 81S and 151S, respectively (Figure 4D–I). The sedimentation coefficients obtained at the lower temperature were significantly lower than those obtained at the higher temperature, mainly due to a decrease in the sedimentation velocity caused by an increase in solvent viscosity with a decrease in temperature. Although temperature affects the sedimentation behavior of a substance, its effect on the proportion results is negligible. The ratios of the proportion results obtained at the two temperatures were between 0.86 and 1.09 (Tables S6 and S7).

### 3.5. SV-AUC Analysis of Multiple Batches of EV71 Vaccine Bulks

**Figure 5:**
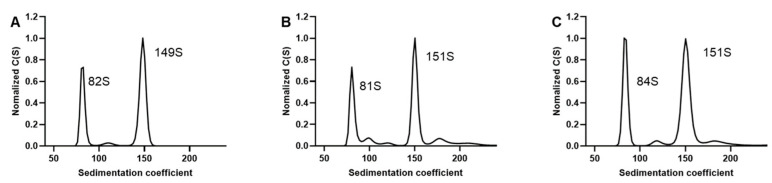
SV-AUC analysis of multiple batches of EV71 vaccine bulks. (**A**–**C**) Sedimentation distribution plots of three lots of EV71 vaccine bulks.

The above findings indicated that SV-AUC has good accuracy and precision in resolving and quantifying EV71 EPs and FPs. To evaluate whether SV-AUC can be used to characterize heterogeneity in EV71 vaccine bulks, three consecutive pilot-scale bulks from an inactivated EV71 vaccine candidate production process were analyzed by SV-AUC. The particles were primarily EPs and FPs (Figure 5), with FPs representing the major species at ~62% across all three lots (Table S8). The consistent genomic particle content across the three bulks demonstrated good process consistency. Thus, SV-AUC can be used to reliably detect and analyze variations in particle distribution resulting from process stability and variability.

## 4. Discussion

The antigens of the licensed EV71 vaccines are obtained from viruses grown in mammalian cells. During multiplication, the virus mainly produces two types of stable particles: EPs and FPs. Similar to that in poliovirus, these two forms of EV71 particles have significant differences in immunogenicity and antigenicity [23,24]. The mature purification process for polio vaccines eliminates all empty viral particles, but current EV71 purification does not separate particle forms, leaving bulk and final vaccine products with both EPs and FPs. This complicates vaccine potency control. The EV71 vaccine antigen detection methods are developed without accounting for differences in the particle form, along with a lack of other analytical discrimination techniques. This results in an inability to effectively evaluate the consistency of particle forms between batches. Therefore, methods to assess the ratio of EPs to FPs need to be developed.

To address this issue, we evaluated the ability of AUC to characterize the ratio of EPs to FPs in inactivated EV71 vaccines. The results showed that AUC could distinguish empty and full particles based on their sedimentation behavior. The SEDFIT software processes absorbance and IF data to generate a C(S) distribution relating sedimentation coefficient values in Svedberg units. By integrating the area under each peak in the C(S) distribution, the relative concentration of each peak was determined to calculate the relative proportion of different particle species in the sample [29]. Our validation showed that this method had high precision. The results of the AUC analysis showed a strong linear correlation between the measured and expected values with a slope close to 1. However, when the theoretical FP proportion exceeded 50%, the FP proportion detected by SV-AUC was slightly higher than the theoretical value, with differences ranging from 11.46% to 19.28% [31] (Table S5). The results obtained by ultraviolet (230 nm) and IF detection methods showed a correlation coefficient of 0.9943. The IF detection data had a direct proportional relationship with concentration and were unaffected by differences in particle absorbance. Therefore, we speculated that the primary reason for the high FP detection results was that the EPs purified by CsCl density gradient centrifugation contained some residual FPs, which led to higher FP proportions in the prepared samples.

Picornaviruses cultured in mammalian cells generate two main types of stable particles as described above: highly infectious mature virions (containing the RNA genome) and empty particles (lacking the RNA genome) [19,20,21,22]. A third type of particle, called the A-particle or uncoating intermediate, is also found [19]. The A-particle has lost its pocket factor, which allows the pocket to collapse and leads to expanded capsids [22,24]. In addition to EPs, FPs, and A-particles, there are some other transitional types of particles. As these particles were scarce and unstable, their proportion was not evaluated in this study.

Like polio vaccines, EV71 inactivated vaccine manufacturing processes can be optimized to selectively retain full particles while removing empty particles via anion-exchange chromatography [42]. A new technique needs to be developed in which SV-AUC may be used as an evaluation method for optimizing the process.

In this study, we investigated the ability of SV-AUC to differentiate between the EPs and FPs of EV71. In this context, our study had some limitations. First, particle forms with partial or fragmented genomes generated during cultivation/purification were not evaluated. Additionally, while evaluating the feasibility of the technique, rigorous validation was not performed. Some aspects like the robustness to variables, such as time, temperature, concentration, and purity, and the comparison of methods to accurately determine performance versus alternate techniques for quantifying particle ratios were not investigated. To achieve a mature detection method, more studies are required to fully assess the ability of SV-AUC to characterize enterovirus particles, such as defining the linear range correlating with the EP-to-FP ratio, determining the specific experimental parameters of the method, and investigating the potential influences of different adjuvants, stabilizers, and preservatives in vaccines. SV-AUC is a promising method for distinguishing different EV71 particle forms. With further optimization and validation, SV-AUC may be used in enterovirus vaccine development, stability studies, and quality control and also, it could be used for other enterovirus-related picornaviruses, e.g., parechovirus (an emerging pediatric pathogen) [43].

## Data Availability

The data presented in this study are available in [[Figshare] at [https://doi.org/10.6084/m9.figshare.24906153]. The methods for the purification of EV71 vaccine bulks are confidential information of the Wuhan Institute of Biological Products Co., Ltd., and they will not be shared. The data presented in this study are available on request from the corresponding author.

## Supplementary Materials

The following supporting information can be downloaded at https://www.mdpi.com/article/10.3390/v16040573/s1, Table S1: The relative percentage of EPs and FPs was determined by the three detection wavelengths; Table S2: A comparison of the relative percentage of EPs, as determined by the ultraviolet (230 nm) and IF detection methods; Table S3: A comparison of the relative percentage of FPs, as determined by the ultraviolet (230 nm) and IF detection methods; Table S4: The precision verification results of the relative percentage of EPs and FPs; Table S5: A summary of the FP content determined by the ultraviolet and IF detection methods compared to the theoretical percentage; Table S6: A comparison of the relative percentage of EPs, as determined at 4 °C and 20 °C; Table S7: A comparison of the relative percentage of FPs, as determined at 4 °C and 20 °C; Table S8: A comparison of the relative percentage of FPs in three lots.
