# Supplementary material for: Quantitation of Enterovirus A71 Empty and Full Particles by Sedimentation Velocity Analytical Ultracentrifugation
Source: Viruses. 2024 Apr 8;16(4):573. doi: 10.3390/v16040573 (PMC11053756; doi:10.3390/v16040573)
Supplement: Supplementary file 1 [file viruses-16-00573-s001.zip › viruses-2929409-supplementary.pdf]

Supplementary information

Quantitation of Enterovirus A71 Empty and Full Particles by Sedimentation Velocity Analytical Ultracentrifugation

Anna Yang[1,2,3,4] Yun Luo[5], Jie Yang[1,2,3,4], Tingbo Xie[1,2,3,4],Wenhui Wang[1,2,3,4], Xin Wan[1,2,3,4], Kaiwen Wang[1,2,3,4], Deqin Pang[1,2,3,4], Dongsheng Yang[1,2,3,4], Hanyu Dai1[1,2,3,4], Jie Wu[1,2,3,4], Shengli Meng[1,2,3,4], Jing Guo[1,2,3,4], Zejun Wang[1,2,3,4], Shuo Shen[1,2,3,4*]

1Wuhan Institute of Biological Products Co. Ltd., No. 1 Huangjin Industrial Park Road, Wuhan 430207, China; 13163255896@163.com(J.Y.); 18995586191@163.com (S.M.)

2National Engineering Technology Research Center of Combined Vaccines, No. 1 Huangjin Industrial Park Road, Wuhan 430207, China

3National Key Laboratory for Novel Vaccines Research and Development of Emerging Infectious Diseases, No. 1 Huangjin Industrial Park Road, Wuhan 430207, China

4Hubei Provincial Vaccines Technology Innozation Center, No. 1 Huangjin Industrial Park Road, Wuhan 430207, China

5The Research Core Facilities for Life Science (HUST), College of Life Science and Technology, Huazhong University of Science and Technology, Luoyu Road, Wuhan 430074, China

*Correspondence: shenshuo0227@163.com

Table S1. The relative percentage of EP and FP was determined by the three detection wavelengths

	230 nm			260 nm			280 nm		
	c-value	S	Capsid content	c-value	S	Capsid content	c-value	S	Capsid content
EP	0.9199	80	49.27%	0.1580	80	29.25%	0.1086	80	11.71%
FP	0.9472	152	50.73%	0.3821	153	70.75%	0.8186	151	88.29%

Table S2. Comparison of the relative percentage of EP, as determined by the ultraviolet (230 nm) and IF detection methods

	230 nm			IF			230 nm/IF
	S	c-value	Content (%)	S	c-value	Content (%)	
EP	82	0.0728	9.76%	80	0.0226	11.24%	0.87
	80	0.2366	36.13%	80	0.0747	39.54%	0.91
	80	0.3896	67.71%	83	0.1331	73.25%	0.99

Table S3. Comparison of the relative percentage of FP, as determined by the ultraviolet (230 nm) and IF detection methods

	230 nm			IF			230 nm/IF
	S	c-value	Content (%)	S	c-value	Content (%)	
FP	151	0.6734	90.24%	149	0.1784	88.76%	1.02
	151	0.4182	63.87%	149	0.1142	60.46%	1.06
	151	0.1858	32.29%	159	0.0486	26.75%	1.02

Table S4. Precision verification results of the relative percentage of EP and FP

	EP			FP		
Repeat	c	Content	S	c	Content	S
1	0.2998	41.32%	83	0.4257	58.68%	147
2	0.2981	41.70%	85	0.4167	58.30%	149
3	0.2653	40.97%	82	0.3822	59.03%	147
4	0.2809	41.13%	82	0.4021	58.87%	143
5	0.2681	47.73%	85	0.2936	52.27%	151
6	0.2673	42.17%	81	0.3666	57.83%	148
Mean	0.2799	42.50%	83	0.38115	57.50%	148
CV	5.62%	6.11%	2.02%	12.62%	4.52%	1.81%

Table S5. A summary of the FP content determined by the ultraviolet and IF detection methods compared to the theoretical percentage

Theoretical % FP	% FP by 230 nm	% FP by IF	%230 nm-%IF
100.00%	100.00%	100.00%	0.00%
85.26%	96.72%	89.48%	7.24%
69.20%	88.48%	84.93%	3.55%
51.65%	69.70%	66.46%	3.24%
32.39%	33.57%	35.47%	-1.90%
11.15%	10.06%	11.14%	-1.08%

Table S6. A comparison of the relative percentage of EP, as determined at 4 °C and 20 °C

	4 °C			20 °C			4 °C/20 °C
	S	c-value	Content (%)	S	c-value	Content (%)	
EP	51	0.0882	9.21%	82	0.0728	9.76%	0.94
	53	0.295	39.36%	80	0.2366	36.13%	1.09
	51	0.5162	72.27%	80	0.3896	67.71%	1.06

Table S7. A comparison of the relative percentage of FP, as determined at 4 °C and 20 °C

	4 °C			20 °C			4 °C/20 °C
	S	c-value	Content (%)	S	c-value	Content (%)	
FP	95	0.8694	90.79%	151	0.6734	90.24%	1.01
	96	0.4545	60.64%	151	0.4182	63.87%	0.95
	96	0.1981	27.73%	151	0.1858	32.29%	0.86

Table S8. A comparison of the relative percentage of FP in three lots

	EP			FP		
	c-value	Content (%)	S	c-value	Content (%)	S
Lot 1	0.2149	36.35%	82	0.3763	63.65%	149
Lot 2	0.2197	38.95%	81	0.3444	61.05%	150
Lot 3	0.1676	38.95%	84	0.2627	61.05%	151
